# Quantifying skin microvascular function responses to distinct forms of heat stress in humans using optical coherence tomography

**DOI:** 10.1113/EP093602

**Published:** 2026-01-28

**Authors:** Kristanti W. Wigati, Robert A. McLaughlin, Harrison T. Caddy, Howard H. Carter, Louise H. Naylor, Daniel J. Green

**Affiliations:** ^1^ School of Human Sciences (Exercise and Sport Science) The University of Western Australia Perth Western Australia Australia; ^2^ Medical Physiology and Biochemistry Department, Faculty of Medicine Universitas Airlangga Surabaya Indonesia; ^3^ School of Biomedicine, Faculty of Health and Medical Sciences The University of Adelaide Adelaide South Australia Australia; ^4^ School of Engineering The University of Western Australia Perth Western Australia Australia; ^5^ Vascular Engineering Laboratory, Harry Perkins Institute of Medical Research Queen Elizabeth II Medical Centre Nedlands Western Australia Australia

**Keywords:** heat exposure, optical coherence tomography, skin microvasculature

## Abstract

Optical coherence tomography (OCT) enables visualization and quantification of the cutaneous microvasculature, yet no study has compared responses to distinct forms of heating in humans. We hypothesized that local skin heating (LH) would evoke larger responses in microvascular diameter, velocity, flow and density than passive whole‐body heating (PH) or heated exercise (HE), and that HE responses would exceed PH. Twelve healthy young adults completed four interventions: baseline (33°C; BL), LH, PH (seated) and HE (ergometer cycling) in a climatic chamber (50 min, 40°C, 50% relative humidity). OCT was used to quantify microvascular variables immediately after each intervention. Microvascular responses differed across conditions (*P *< 0.001). LH induced the largest responses in all OCT indices (all *P *< 0.001): diameter (67 µm), velocity (195 µm s^−1^), flow (687 picolitres s^−1^) and density (56.0%), compared with BL (42 µm, 106 µm s^−1^,154 picolitres s^−1^ and 26.6%, respectively), PH (45 µm, 99 µm s^−1^, 165 picolitres s^−1^ and 34.4%, respectively) and HE (49 µm, 105 µm s^−1^, 208 picolitres s^−1^ and 34.5%, respectively). Although the diameter response was higher after HE (*P *= 0.046), no differences were documented for PH and HE relative to 33°C BL for other OCT measures (all *P *> 0.05). Comparable responses were observed between PH and HE across all variables (all *P *> 0.05). Local heating elicited substantially greater increases in all OCT‐derived microvascular metrics compared with PH and HE. Although both PH and HE activate the cutaneous microvasculature, neither stimulus approaches the magnitude of response achieved with local heating. These findings demonstrate that OCT provides quantifiable insights into the distinct ways in which the skin microvasculature responds to different heat exposures.

## Abstract (highlights)

- **What is the central question of this study?**
  Do different thermal stimuli (33°C baseline, local skin heating, passive whole‐body heating and heated exercise) elicit distinct patterns of response in cutaneous microvascular diameter, flow and density in humans, quantified using optical coherence tomography?
- **What is the main finding and its importance?**
  Local heating elicited substantially greater increases than other heating stimuli in all optical coherence tomography‐derived metrics. Whole‐body heating and heated exercise each activate the cutaneous microvasculature, but neither stimulus approached the magnitude of response observed with local heating. These findings improve our understanding of how distinct thermal challenges engage cutaneous effector mechanisms, with implications for thermoregulatory reserve and heat tolerance in varied physiological and clinical contexts.

## 1 INTRODUCTION

The skin is a principal effector organ for human thermoregulation. A dense and specialized architectural network of cutaneous microvessels, with a remarkable capacity to increase blood flow (to ∼8 L min^−1^; Rowell, 1974), facilitates heat loss from the skin surface (Johnson et al., 2014). Thermal stress can arise from the environment or endogenously via heat generated by skeletal muscle during dynamic exercise. External (passive) heat stress induces redistribution of blood flow from visceral circulations (e.g., splanchnic, renal and resting muscle) via sympathetic vasoconstriction (Rowell, 1974). When exercise is added to passive heat stress, increases in metabolic demand in the active muscle compete for cardiac output, with increases in skin perfusion to subserve thermoregulation (Johnson, 2010; Rowell, 1983). Consequently, exercise increases the core temperature (*T*_c_) threshold for skin vasodilatation, and increases in skin blood flow are attenuated with respect to *T*_c_ as exercise progresses (Brengelmann et al., 1977; Johnson, 1986). Localized skin heating, achieved by applying a heating patch to the skin surface, can also induce cutaneous vasodilatation, which reflects localized and some neural reflex changes in microvascular function (Minson, 2010). This thermal hyperaemia test has been used to understand the progression of microvascular disease and responses to treatments and interventions (Brunt & Minson, 2011).

Historical assessment of skin blood flow has relied upon approaches such as plethysmography, which estimates relative changes in whole‐limb blood flows (Argarini et al., 2020), or laser Doppler flowmetry (LDF) (Black et al., 2008; Green et al., 2006) which reflects relative changes in red cell flux. These approaches are qualitative in nature and do not directly visualize or quantify microvascular parameters. This limits our understanding of the nature of change in blood flow to the skin, for example, whether smaller vessels are recruited and vascular ‘density’ increases, or whether changes occur in diameter, velocity, flow or a combination of these.

We recently developed a high‐resolution non‐invasive imaging technique based on cutaneous optical coherence tomography (OCT), which uses low‐power near‐infrared light and is capable of visualizing and quantifying blood vessel diameter, velocity, perfusion density and flow, in vessels as small as 30 µm in diameter (Smith et al., 2019). Using this approach, we have shown that OCT is feasible and reliable for visualizing and quantifying microvascular structure and function in healthy volunteers and individuals with type 2 diabetes (Argarini et al., 2020) and heart failure (Sciarrone et al., 2022), that it can distinguish disease severity (Argarini et al., 2020) and quantify the impact of exercise training on cutaneous vessels (Argarini et al., 2021). These studies have relied upon physiological stimulation of small regions of skin beneath or adjacent to the OCT probe (i.e., ‘local’ heating). Studies using OCT to characterize changes in the response to systemic heating and/or exercise have not previously been reported or compared with those achieved using localized heating. In the present study, we used OCT to quantify cutaneous microvascular responses to local skin heating (LH) and compared these with measures obtained in response to passive whole‐body heating (PH) in a heat chamber at rest and during cycling exercise (HE). We hypothesized that: (1) LH would induce larger responses in indices of cutaneous microvascular diameter, velocity, flow and density than those observed in response to PH and HE; and (2) that HE would induce larger skin microvascular responses than those obtained in response to PH.

## 2 MATERIALS AND METHODS

This study complied with the *Declaration of Helsinki*, and the protocol was reviewed and approved by the Human Research Ethics Committee of the University of Western Australia (ref. RA/4/20/5716). All participants provided written informed consent before their involvement in this study.

### 2.1 Subjects

Healthy young male and female participants were recruited from the local Perth community, Western Australia. They were screened for exclusion criteria, including any history of cardiovascular disease (e.g., myocardial infarction, stroke, diabetes), implanted devices (e.g., pacemakers), hypertension (resting systolic pressure >160 mmHg or diastolic pressure >100 mmHg), smoking, obesity (body mass index >35 kg m^−2^), low body weight (<37 kg) or any regular medications.

### 2.2 Study design

Before commencing the whole‐body heating protocol while resting or exercising, participants were invited to undertake baseline assessments, which included body height, weight, standardized 33°C baseline (BL), local skin heating (LH) and peak oxygen consumption (${\dot{V}}_{O_{2}\mathrm{peak}}$) testing.

#### 2.2.1 Baseline ${\dot{V}}_{O_{2}\mathrm{peak}}$ testing

Participants performed an initial ${\dot{V}}_{O_{2}\mathrm{peak}}$ test (TrueOne 2400, Parvo Medics, Salt Lake City, UT, USA) on a stationary cycle ergometer (Excalibur Sport, Lode BV, Groningen, The Netherlands). This test consisted of a 3 min warm‐up phase cycling at 50 W, followed by 25 W increments in resistance per minute until their maximal effort and the participant voluntarily terminated the test. They were instructed to maintain a cadence between 50 and 70 r.p.m., and heart rate (HR) was monitored continuously (Polar H10, Polar, Kempele, Finland) throughout the test. Gas analysers were calibrated before each test using room air and a certified gas mixture (4% CO_2_, 16% O_2_, balance N_2_; Airgas Healthcare, Miami, FL, USA), and ${\dot{V}}_{O_{2}\mathrm{peak}}$ was normalized by body weight (in millilitres per kilogram per minute).

#### 2.2.2 Standardized 33°C baseline and localized skin heating assessment

The LH assessment was conducted on the same day as the 33°C standardized baseline (BL) assessments, before participants were then invited to carry out PH and HE. Prior to the LH intervention, participants were asked to fast for 8 h and refrain from exercise for 24 h and from coffee, tea or chocolate for 12 h. The skin sites for microvascular assessment were shaved 24 h before the assessment (if required). OCT was used to visualize and quantify the skin microvasculature non‐invasively, as described in previous studies (Carter et al., 2016; Sciarrone et al., 2022), with a brief technical summary provided below. A commercial imaging system (Telesto III, Thorlabs, Germany) was used to acquire a three‐dimensional image over a 5 mm × 5 mm × 2.5 mm field of view selected on the ventral side of the forearm. The OCT light beam was weakly focused, allowing high spatial resolution (13 µm beam waist) to a depth of 1 mm below the skin surface (Argarini et al., 2020). A bespoke optical spacer was placed rigidly between the OCT probe and the skin to ensure that the beam waist was positioned 300 µm below the tissue surface. A small droplet of water was placed between the skin and the optical window of the spacer to reduce artefacts from refractive index mismatches. A sequence of 10 co‐located OCT B‐scans were acquired at each position across the field of view, with a spacing of 10 µm between sets of B‐scans. Total acquisition time across the field of view was 90 s. This acquisition time was found to be well tolerated by participants, minimizing movement artefact (Argarini et al., 2020). In OCT imaging, movement gives rise to measurable changes in the speckle noise present in the optical signal. Blood flow was detected and quantified by measuring the rate of change of speckle noise at each location within each set of 10 co‐located B‐scans. Analysis software developed in‐house in MATLAB (v.2022b) was then used to quantify median diameter, median blood velocity, median flow and the density of the skin microvessels of the forearm. Density was calculated by generating a two dimensional maximum intensity projection (MIP) image in the *X*–*Y* plane (parallel to the skin surface), where each pixel value corresponds to the maximum flow speed at the (*x*, *y*) location taken over all depths (*z*). Speeds were thresholded at the minimum detectable speed, which was 58 µs^−1^ for the acquisition parameters used in this specific study. The density was computed as the percentage of pixels with detectable flow, consistent with earlier validation studies using OCT to assess the structural and functional metrics of skin microvasculature for local heating (Smith et al., 2019). Prior to BL and LH scans, the skin was locally heated to standardized temperatures using a local heating disk (PF450, Perimed, Stockholm, Sweden). In a quiet and temperature‐controlled room, 33°C standardized BL data were collected after 20 min of quiet rest, with skin temperature under the heating disk maintained at 33°C. This baseline skin temperature was adopted to ensure a thermoneutral standardization unlikely to elicit significant vasodilatation or constriction (Charkoudian, 2010; Johnson & Kellogg, 2010). A local heating scan was then obtained after 30 min of skin disk heating at 44°C (Argarini et al., 2021). While OCT data were collected, simultaneous recordings of red blood cell flux were obtained using LDF that was heated using a second heating disk, using an identical protocol. OCT and LDF probe holders were co‐registered adjacent to each other (distance of <5 cm) on the same anatomical region (ventral forearm) and were affixed using double‐sided adhesive rings (Argarini et al., 2021). The skin flux was captured and analysed across the same time period as that used to obtain the OCT images.

#### 2.2.3 Whole‐body passive chamber heating, combined with cycle ergometer exercise

After completion of the baseline assessments, the participants were then invited to undertake two separate experimental sessions on separate days, involving either passive whole‐body heating in a climate chamber (PH) or exercise in a heated climate chamber (HE). The order of these interventions was randomized and counterbalanced using a coin toss. Each session was separated by ≥48 h to allow a washout period but performed at the same time of day to minimize circadian variation. Before undergoing either session, participants were asked to refrain from consuming coffee, tea or chocolate for 12 h, or from vigorous exercise within 24 h. As described above, the skin sites used for microvascular assessments were shaved 24 h before the assessment (Argarini et al., 2020). Post‐intervention assessments were recorded inside the heat chamber immediately after the completion of the PH or HE exposure.

For the PH session, participants remained seated in an upright position in a chair placed in a climate‐controlled chamber for 50 min, at 40°C, 50% relative humidity (RH) (wet bulb temperature = 30.9°C). The HE session involved cycling on a stationary cycle ergometer (Ergomedic 828 E, MONARK, Vansbro, Sweden) at 45% ${\dot{V}}_{O_{2}\mathrm{peak}}$, for 50 min, in the same environmental conditions (40°C, 50% RH, wet bulb temperature = 30.9°C). This was reduced in 5 W increments at 5 min bands of testing as required to maintain HR at ≤80% of peak HR (Caddy et al., 2024). During both interventions inside the heat chamber, thermoneutral water was provided ad libitum. If, at any point, the *T*_c_ of the participant exceeded 39.0°C or if they exhibited signs or symptoms of heat‐related illness, participants were immediately removed from the heat chamber.

### 2.3 Experimental assessments

#### 2.3.1 Core temperature

The *T*_c_ was monitored throughout the intervention protocols using an ingested radiotelemetry gastrointestinal pill and monitor (eCelcius Performance; BodyCap Medical, Hérouville‐Saint‐Clair, France). The participants were asked to ingest the pill ∼6 h before the experiment to ensure an ideal location within the gastrointestinal tract (Domitrovich et al., 2010). HR was monitored continuously using a wearable HR sensor (Polar H10), and mean arterial pressure (MAP) was measured manually using a stethoscope and pneumatic arm cuff.

#### 2.3.2 Skin microcirculation

**FIGURE 1:**
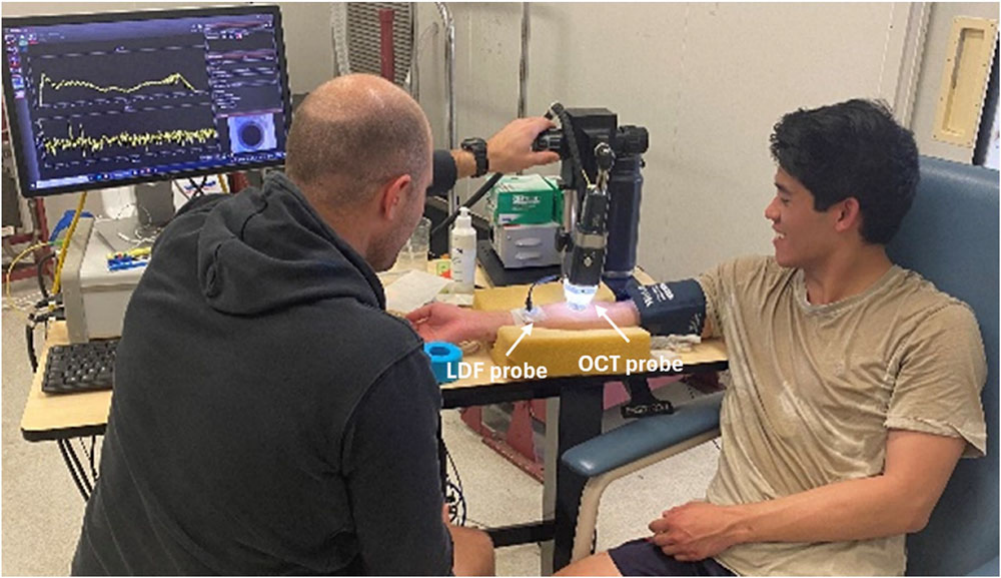
Instrumentation during the assessment of skin microvessels using OCT and LDF concurrently inside the controlled climate chamber following 50 min of either PH or HE. The OCT probe is attached to the skin adjacent to the LDF probe (<5 cm) and is affixed using double‐sided tape. Abbreviations: HE, heated exercise; LDF, laser Doppler flowmetry; OCT, optical coherence tomography; PH, passive whole‐body heating.

Skin blood flow was assessed immediately after each 50 min PH and HE intervention while participants remained inside the heat chamber. OCT was used to acquire images from the ventral side of the forearm (Figure 1), which was shaved 24 h before the visit. The OCT probe contact location on the forearm was marked to retain the same spatial location between tests and visits.

For comparative purposes (with OCT data), we simultaneously recorded red blood cell flux using laser Doppler flowmetry (LDF, model 413, Periflux 5000 System; Sweden), with integrated probes located adjacent to the OCT probe (as shown in Figure 1). The probe was attached to the skin using double‐sided adhesive tape, and data were collected using LabChart v.7 (AD Instruments, Sydney, NSW, Australia) and averaged over the same time window as that used to obtain the OCT images (90 s).

### 2.4 Data collection and analysis

The sample size calculation was based on the published data of Smith et al. (2019), who reported the reproducibility pertaining to the quantitative OCT technique we have developed, including a comparison with the reproducibility of the traditionally used LDF approach. Their study indicated that the coefficient of variation for within‐subject between‐day OCT‐derived measures of diameter (14%) and velocity (10%) were both substantially lower than measures derived from laser Doppler (LD) flux assessment (23%). Using these data, and assuming 80% power and α = 0.05, the differences in responses we observed to local heating in the present study, versus those observed in response to passive heating, indicate that a sample size of five subjects was adequate to detect a significant difference between these interventions. Applying similar approaches to the difference in responses we observed between local heating and heated exercise, a sample size of six subjects was adequate to detect a significant difference between the interventions. Data were normally distributed for each intervention as assessed by Shapiro–Wilk test (all *P *> 0.05), and there was homogeneity of variances as assessed by Levene's test (all *P *> 0.05). All data are the means ± SD across subjects, unless otherwise stated. A two‐way repeated‐measures ANOVA was used to analyse differences in core temperature and haemodynamic responses between interventions. A one‐way ANOVA was performed to compare OCT‐ and LDF‐derived microvascular responses between interventions. When ANOVA tests were significant, *post hoc* analysis using Tukey's test was applied. Statistical significance was assumed at *P* < 0.05.

## 3 RESULTS

Twelve young healthy participants (six males and six females; age, 25.9 ± 3.6 years; body mass index, 22.5 ± 2.2 kg m^−2^) enrolled in the study following screening for exclusion criteria and providing written informed consent. The menstrual cycle was not controlled experimentally; however, it was recorded for all female participants. Of the six female participants, four were actively taking oral contraceptives, and the remaining two were tested within the follicular and luteal menstrual cycle phases, respectively.

The peak HR documented from the cycle exercise test was 178 ± 8 beats min^−1^, and ${\dot{V}}_{O_{2}\mathrm{peak}}$ was 44.4 ± 11.2 mL kg^−1^ min^−1^. Owing to movement artefact (*n* = 2), a hyperthermic response to the intervention (*n* = 1) and core temperature pill failure (*n* = 1), complete standardized 33°C BL and post‐intervention data are presented in eight participants.

### 3.1 PH and HE impacts on core temperature and haemodynamics

**FIGURE 2:**
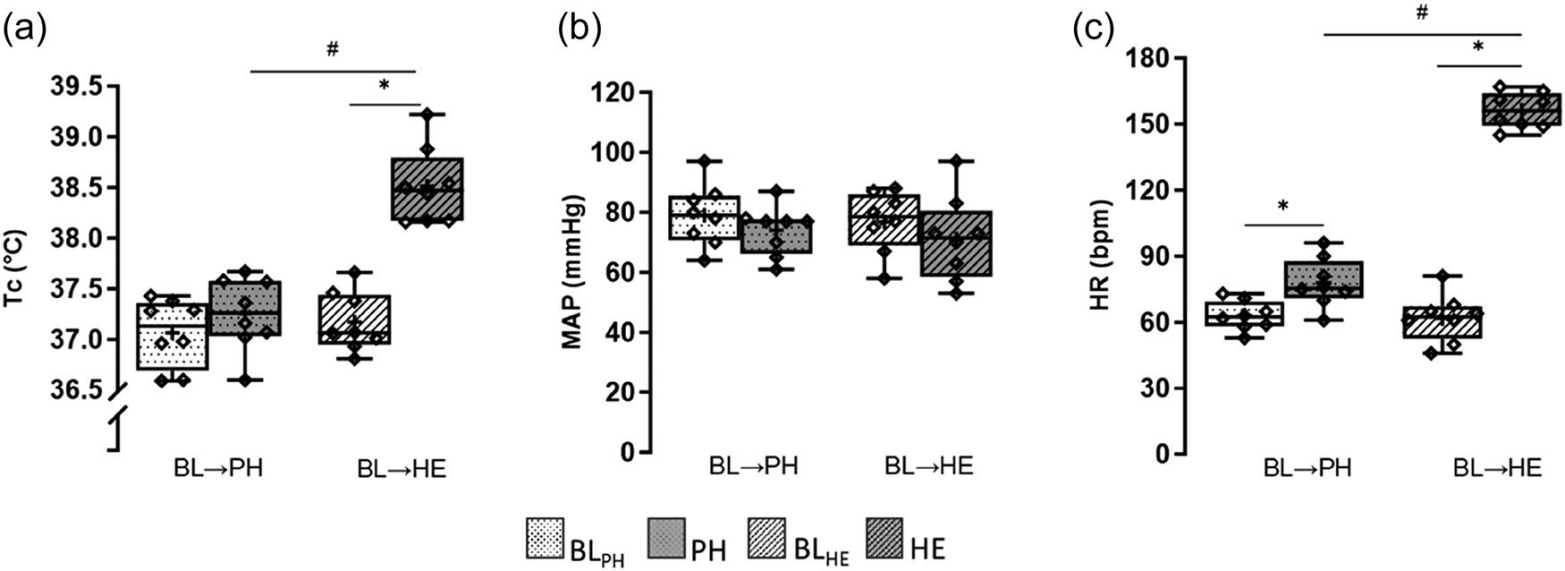
Core temperature (*T*_c_; a), mean arterial pressure (MAP; b) and heart rate (HR; c) were measured before (baseline; BL) and after 50 min bouts of passive whole‐body heating (PH) and heated exercise (HE). (**+**) Mean; *P < 0.05 for time effects; ^#^ *P* < 0.001 for interaction effects.

Core temperature increased significantly after HE when compared with baseline, but this was not the case after PH (ANOVA time effect, *P* < 0.001; Figure 2a). There was a significant interaction apparent between PH and HE (*P* < 0.001) for the change in *T*_c_; *post hoc* testing revealed that *T*_c_ increased significantly following HE (37.1°C ± 0.3°C vs. 38.5°C ± 0.3°C, *P* < 0.001), but was not significantly impacted by PH (37.0°C ± 0.3°C vs. 37.2°C ± 0.3°C, *P* = 0.258).

For MAP (PH: 79 ± 10 vs. 74 ± 8 mmHg, *P* = 0.182; HE: 77 ± 10 vs. 71 ± 14 mmHg, *P* = 0.138), no significant time effect was evident (*P* = 0.054; Figure 2b), and there was no interaction between the conditions (*P* = 0.907). A time effect was evident for HR (*P* < 0.001; Figure 2c), with a significant interaction apparent between PH and HE (*P* < 0.001). *Post hoc* testing revealed that HR increased significantly higher after PH (63 ± 7 vs. 78 ± 11 beats min^−1^, *P* = 0.006) and HE (62 ± 11 vs. 156 ± 8 beats min^−1^, *P *< 0.001).

### 3.2 Standardized 33°C BL, LH, PH and HE impacts on OCT‐derived microvascular diameter, velocity, flow and density

**FIGURE 3:**
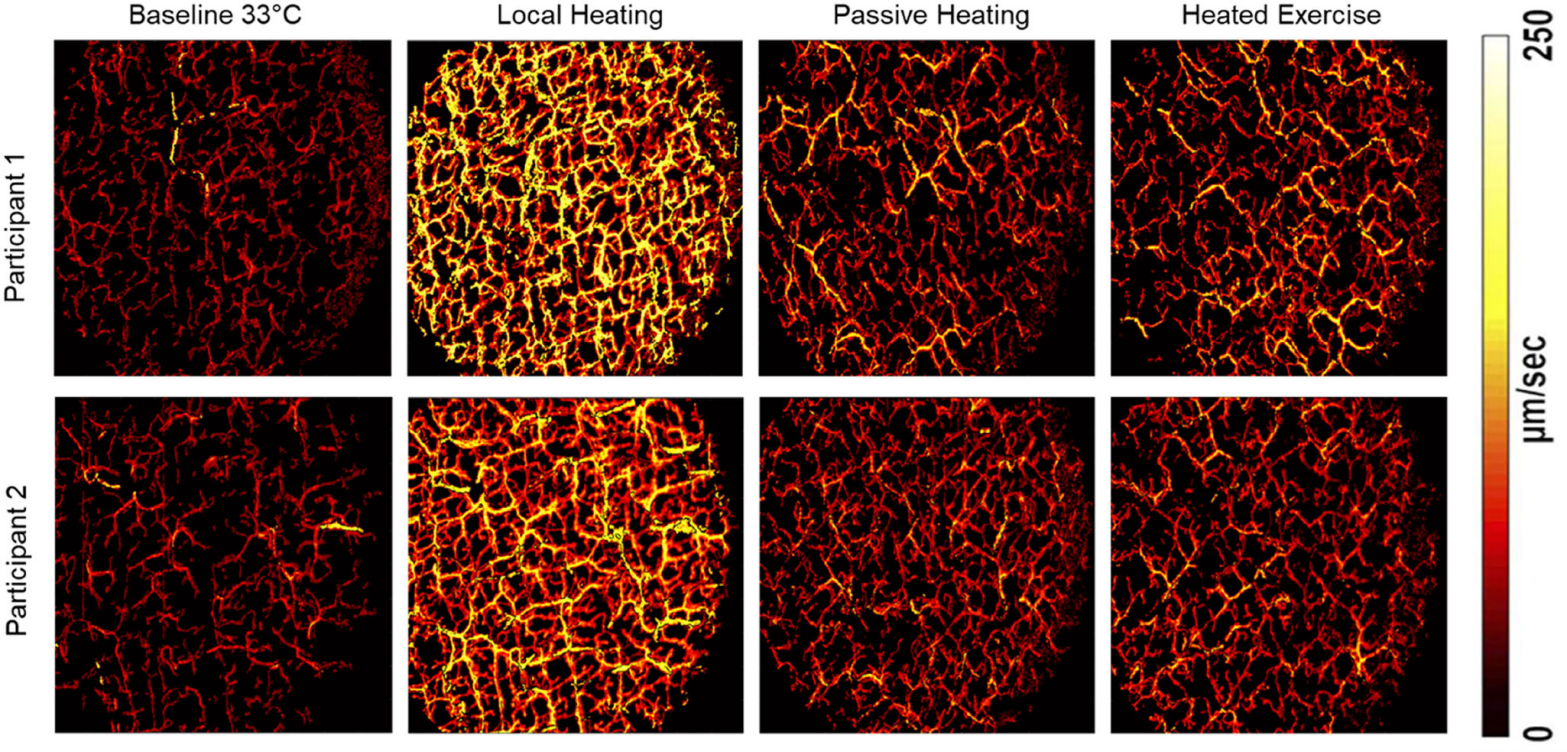
Representative optical coherence tomography‐derived images from two participants in response to standardized 33°C BL, and following 30 min of LH, 50 min of PH or HE. Blood vessels are colour‐coded to indicate blood velocity (in micrometres per second). Abbreviations: BL, baseline; HE, heated exercise; LH, local heating; PH, passive whole‐body heating.

**FIGURE 4:**
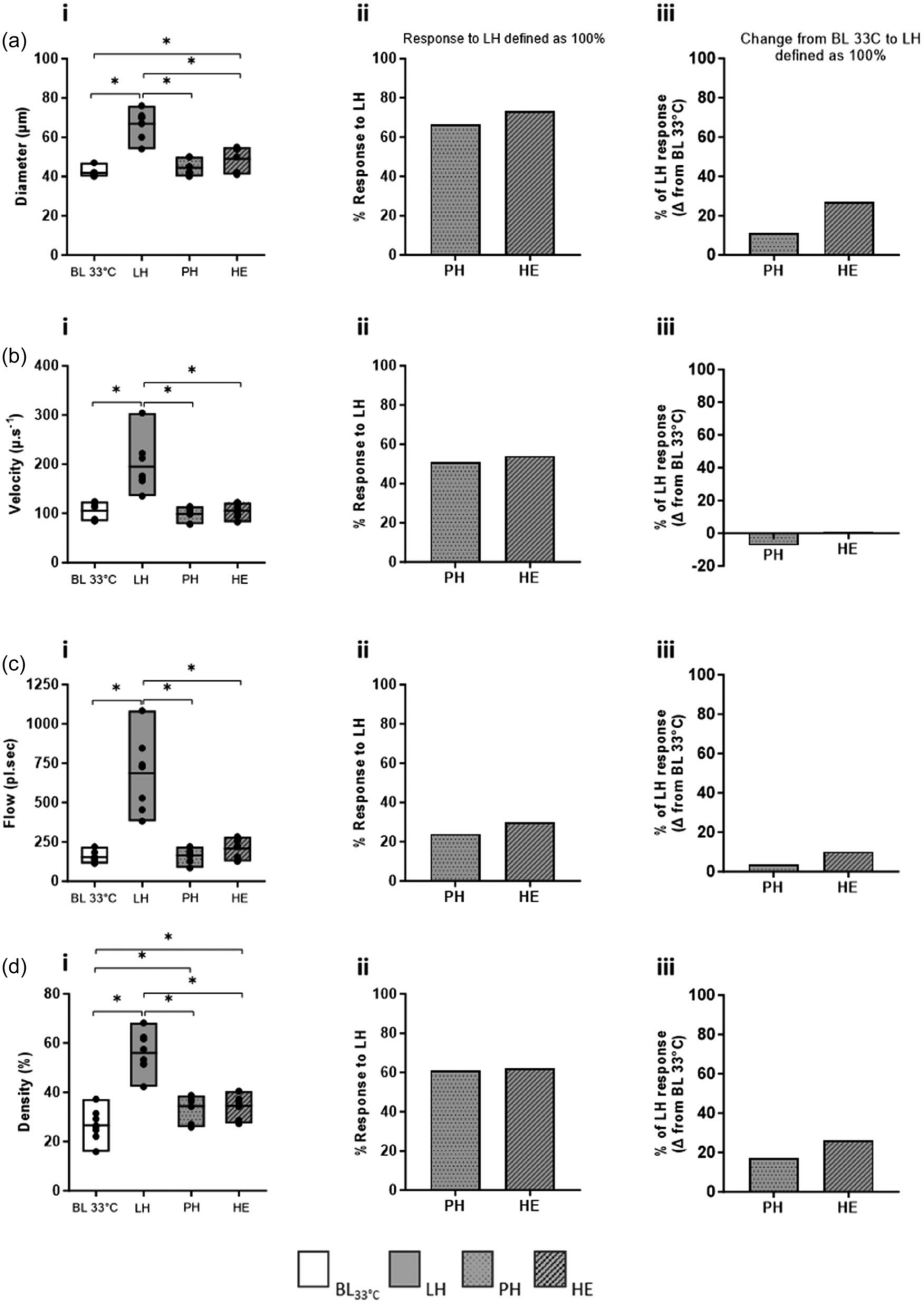
[a(i)–d(i)] OCT‐derived skin microvascular responses, including diameter, velocity, flow and density during standardized 33°C baseline (BL), local heating (LH), passive whole‐body heating (PH) and with exercise (HE). LH elicited significantly larger responses across all OCT‐derived indices than 33°C BL, PH and HE. Relative to 33°C BL, PH and HE stimuli induced similar responses, whereas diameter increases only in response to HE. [a(ii)–d(ii)] The proportion of the heating response to LH when PH and HE are stimuli in absolute terms [e.g. (Mean_PHresponse/Mean_LHresponse) × 100%] and [a(iii)–d(iii)] in the change from standardized 33°C BL [e.g. (Mean_PHresponse − Mean_33°C BL)/(Mean_LHresponse − Mean_33°C BL) × 100%]. *P < 0.05. Abbreviations: BL, baseline; HE, heated exercise; LH, local heating; OCT, optical coherence tomography; PH, passive whole‐body heating.

Figure 3 depicts a representative OCT‐derived image from two participants at standardized 33°C BL and after LH, PH and HE, and the group‐based OCT‐derived median diameter, median velocity, median flow and density response to LH, PH or HE are presented in Figure 4a(i)–d(i). Additionally, we define the absolute peak response [Figure 4a(ii)–d(ii)] and change from BL [Figure 4a(iii)–d(iii)] under LH as 100% vasodilatory reserve, and the proportion of reserve used by PH and HE (expressed as a percentage).

A one‐way ANOVA showed a significant effect of condition on OCT‐derived microvascular diameter [*P *< 0.001; Figure 4a(i)]. *Post hoc* testing revealed that skin microvascular diameter following LH (67 ± 7 µm) was significantly higher than at 33°C BL (42 ± 3 µm; *P *< 0.001), PH (45 ± 4 µm; *P *< 0.001) and HE (49 ± 6 µm; *P *< 0.001). No significant difference was observed between PH and HE (*P *= 0.318) or between 33°C BL and PH (*P *= 0.738). The proportion of the peak heating diameter response to LH when PH and HE were stimuli, in absolute terms, was 67% and 73%, respectively [Figure 4a(ii)]. Additionally, the proportions of PH and HE changes in microvascular diameter from the standardized 33°C BL, compared with LH, were 11% and 27%, respectively [Figure 4a(iii)].

For OCT‐derived microvascular velocity, a one‐way ANOVA showed a significant effect of intervention [*P *< 0.001; Figure 4 c(i)]. *Post hoc* tests revealed that skin microvascular velocity following LH (195 ± 52 µm s^−1^) was significantly higher than 33°C BL (106 ± 17 µm s^−1^; *P *< 0.001), PH (99 ± 14 µm s^−1^; *P *< 0.001) and HE (105 ± 14 µm s^−1^; *P *< 0.001). No significant difference was observed between the response of 33°C BL and PH (*P *= 0.966), 33°C BL and HE (*P *= 1.000) or PH and HE (*P *= 0.969) The proportion of the peak heating diameter response to LH when PH and HE were stimuli, in absolute terms, was 51% and 54%, respectively [Figure 4 c(ii)]. Additionally, the proportions of PH and HE changes in microvascular diameter from the standardized 33°C BL, compared with LH, were −7.56% and −0.28%, respectively [Figure 4c(iii)].

In terms of cutaneous OCT‐derived blood flow, a one‐way ANOVA also showed a significant effect of intervention [*P *< 0.001; Figure 4 c(i)]. *Post hoc* analysis indicated that blood flow following LH (687 ± 227 pl s^−1^) was significantly higher than 33°C BL (154 ± 33 pl s^−1^; *P *< 0.001), PH (165 ± 42 pl s^−1^; *P *< 0.001) and HE (208 ± 60 picolitres s^−1^; *P* < 0.001). No significant differences were observed in response to 33°C BL versus PH (*P *= 0.998), versus HE (*P *= 0.805) and PH versus HE (*P *= 0.889). The proportions of the peak heating blood flow response to LH when PH and HE were stimuli, in absolute terms, were 24% and 30%, respectively [Figure 4c(iii)]. Additionally, the proportions of PH and HE changes in blood flow from the standardized 33°C BL, compared with LH, were 4% and 10%, respectively [Figure 4c(iii)].

Finally, a one‐way ANOVA showed a significant effect of intervention on OCT‐derived microvascular density [*P *< 0.001; Figure 4d(i)]. *Post hoc* testing showed that microvascular density following LH (56.0% ± 8.1%) was significantly higher than 33°C BL (26.8% ± 6.8%; *P *< 0.001), PH (34.4% ± 5.1%; *P *< 0.001) and HE (34.5% ± 4.4%; *P *< 0.001). Additionally, a significant difference was observed in response to BL versus PH (*P *= 0.044) and BL versus HE (*P *= 0.040), but not PH versus HE (*P *= 1.000). The proportions of the peak heating microvascular density response to LH when PH and HE were stimuli, in absolute terms, were 61% and 62%, respectively [Figure 4d(ii)]. Additionally, the proportions of PH and HE changes in density from the standardized 33°C BL, compared with LH, were 17% and 26%, respectively [Figure 4d(iii)].

**FIGURE 5:**
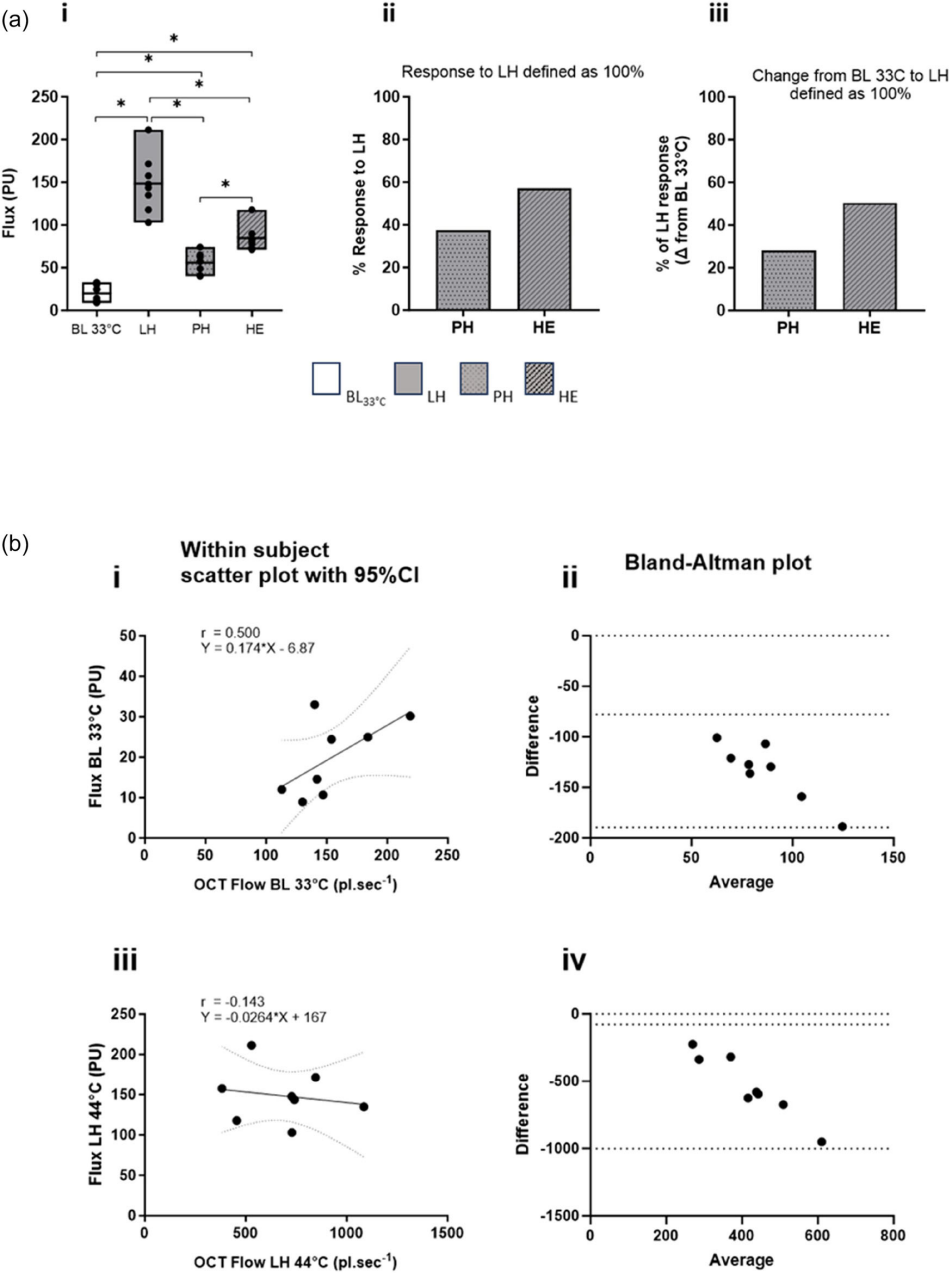
[a(i)] Laser Doppler‐generated forearm skin flux (PU) at 33°C standardized baseline (BL) and following 30 min of local heating (LH) and 50 min of either whole‐body passive heating (PH) or with exercise (HE). LH generated a significantly higher response in blood flux than the responses at 33°C BL, PH and HE. Additionally, skin flux response to HE was significantly higher than that to PH. [a(ii)] The proportion of the peak heating response to LH when PH and HE are stimuli in absolute terms [e.g. (Mean_PHresponse/Mean_LHresponse × 100%] and [a(iii)] in the change from 33°C BL [e.g. (Mean_PHresponse − Mean_33°C BL)/(Mean_LHresponse − Mean_33°C BL) × 100%]. *P < 0.05. [b(i)] The within‐subject scatter plot with 95% confidence interval for OCT‐derived blood flow and LD flux shows modest correspondence. [b(ii)] The Bland–Altman plot analysis demonstrated a non‐agreement between the two techniques in thermoneutral conditions. [b(iii)] The within‐subject scatter plot with 95% confidence interval for OCT‐generated skin microvascular flow shows a larger discrepancy where OCT and LDF are weakly related. [b(iv)] Bland–Altman analysis revealed an even greater negative bias and broader limits of agreement in the LH conditions. Abbreviations: BL, baseline; HE, heated exercise; LDF, laser Doppler flowmetry; LH, local heating; OCT, optical coherence tomography; PH, passive whole‐body heating.

For laser Doppler derived skin flux (calculated as perfusion unit: PU), a one‐way ANOVA showed a significant effect of intervention on blood flux [*P *< 0.001; Figure 5a(i)]. *Post hoc* testing revealed that the blood flux response following LH (148.43 ± 33.41 PU) was significantly higher than 33°C BL (19.85 ± 9.41 PU; *P *< 0.001), PH (55.86 ± 11.87 PU; *P *< 0.001) and HE (84.66 ± 14.69 PU; *P *< 0.001). Moreover, the blood flux response to HE was significantly higher than at 33°C BL (*P* < 0.001) and PH (*P *= 0.006). The proportions of the peak heating blood flux response to LH when PH and HE were stimuli, in absolute terms, were 38% and 57%, respectively [Figure 5a(iii)]. The proportions of PH and HE changes in blood flux from the standardized 33°C BL, compared with LH, were 28% and 50%, respectively [Figure 5a(iii)].

At a standardized 33°C BL, OCT‐derived blood flow and LD flux showed only modest correspondence [*r* = 0.50; Figure 5b(i)], and Bland–Altman analysis demonstrated a large negative bias, with wide limits of agreement [Figure 5b(ii)], indicating clear non‐agreement between the two techniques in thermoneutral conditions. During LH, this discrepancy became more pronounced; OCT and LDF were weakly related [*r* = −0.14; Figure 5b(iii)], and Bland–Altman analysis revealed an even greater negative bias and broader limits of agreement [Figure 5b(iv)]. Taken together, these findings confirm that OCT and LDF provide non‐interchangeable and physiologically distinct measures of skin perfusion, particularly during heating stress.

## 4 DISCUSSION

We aimed to investigate microvascular responses to a standardized 33°C baseline, localized skin heating, resting passive climate chamber heating, and combined exercise and climate chamber heating, using an OCT technique capable of visualizing and quantifying cutaneous microvessel parameters in vivo. LH produced the largest microvascular responses across all OCT variables compared with other heating conditions. The absolute responses of microvascular diameter, flow and density during PH and HE remained well below the levels attained during LH, indicating that a substantial vasodilatory ‘reserve’ remained unused relative to the LH conditions. These findings support our hypothesis that LH would elicit quantitatively larger OCT‐based microvascular responses than PH and HE. Similar responses were documented for PH and HE relative to 33°C BL, whereas diameter was significantly higher only after HE. No significant differences were observed between PH and HE across any OCT parameter. These data reject the proposal that HE induces larger impacts on microvascular responses compared with PH.

Previous studies characterizing responses to whole‐body passive heating and/or heated exercise have relied upon techniques such as laser Doppler flowmetry to assess functional changes in skin microvessels (Fujii et al., 2018, 2017; Kamijo et al., 2005; Kellogg et al., 1998; McGarr et al., 2020; McNamara et al., 2014; Shibasaki et al., 2002). LDF does not generate images of microvessels, nor does it quantify the diameter, flow or vascular recruitment (density). Changes in LDF signals reflect gross movement (flux) of red cells beneath the probe, without characterizing the direction of this movement, or flow through individual vessels. LD flux might therefore, theoretically, change owing to increases in red cell velocity in the presence or absence of concomitant changes in microvascular diameter. Changes in the recruitment and/or redistribution of blood through underlying microvessel beds are also not possible to deduce using LD flux. In the present experiment, we applied LDF using an integrative 7 Doppler array probe to compare our findings with those derived from OCT. We observed that LD flux responses to LH were significantly larger compared with 33°C BL, PH and HE. Historically, these LDF data might have been interpreted as indicating that microvascular diameter and/or flow had increased, without direct assessment of either metric. The insights provided by OCT reveal that flow increased principally owing to changes in diameter in LH and HE conditions, whereas PH induced changes in microvascular density only. Given that the physiological control of microvessels is likely to differ with respect to the determinants of diameter, velocity and recruitment, OCT provides new insights into the mechanisms responsible for changes that occur in response to distinct forms of heat stress.

Notwithstanding the limitations of using LDF to characterize microvascular responses, this approach has provided historical insight into the local and reflex mechanisms responsible for cutaneous microvascular responses to heat exposure. Classic studies revealed a role for NO‐mediated vasodilatation in response to local skin heating, with other transmitters also implicated, namely prostanoids (McCord et al., 2006; Minson et al., 2001), endothelium‐derived hyperpolarizing factor (EDHF) (Choi et al., 2014), neuropeptide Y (NPY) and noradrenaline (Hodges et al., 2008, 2009), and K^+^ channels (Shastry & Joyner, 2002). In our previous studies, we observed adaptation in forearm cutaneous LDF responses following repeated episodic local forearm heating, and also following repeated lower‐limb heating, which induced upper‐limb thermoregulatory vasodilatation (Carter et al., 2014; Naylor et al., 2011). Other studies using microdialysis to block NO production revealed that exercise training reversed the age‐related decline in NO‐mediated vasodilator function in cutaneous microvessels (Black et al., 2008). These studies suggest that, in response to whole‐body passive heating or combined exercise and body heating, heat shock proteins can be upregulated in the skin, alongside augmented nitric oxide (NO) bioavailability, with consequent increases in LDF responses (Fujii et al., 2017, 2017; McGarr et al., 2020; McNamara et al., 2014). In the present study, *T*_c_ increased by ∼0.2°C in response to PH, and microvascular diameters did not increase, yet a significant response was apparent in vessel density, and these responses approximated those observed in response to HE, which substantially raised *T*_c_ and also increased microvascular diameters and density. Additionally, the reflex increase in skin blood flow in response to elevated skin temperature during exercise is augmented as *T*_c_ continues to rise (Johnson & Park, 1979). These findings suggest that, inconsistent with the known control of skin circulation, the systemic response to PH impacts the density of microvessels, larger increases in *T*_c_ and consequent reflex control of the microvessels in response to HE might be necessary to induce changes in microvascular diameter. Additionally, local skin heating to 44°C during the LH protocol induces the largest responses in overall OCT‐based quantifications. Hence, differences exist in the way in which microvessels respond to distinct heating modalities. Future studies using OCT approaches should provide new insights into the mechanisms and effector pathways involved in microvascular adaptations in humans.

We hypothesized that LH would induce larger microvascular responses than PH and HE, on the basis that LH produces near maximum impacts on skin vasodilatation. Each PH and HE stimulus activates the cutaneous microvasculature, but neither approaches the magnitude of responses achieved by LH. PH used ∼67% of the LH‐defined vasodilatory reserve, whereas HE used ∼73%. Our findings support this hypothesis by providing the first quantitative comparison between these interventions in humans. We also hypothesized that HE would induce larger microvascular responses than PH, because exercise generates a larger heat load (*T*_c_ increase) than PH, consequently with larger reflex activation of skin vasodilator mechanisms. This hypothesis was not supported by our observed response in OCT metrics, which were similar in response to HE and PH. One possible explanation for the similar responses between these conditions is that the addition of exercise to passive heating introduced competition for blood flow distribution between the skin and active skeletal muscle beds. This ‘Rowellian’ explanation suggests that human integrative cardiovascular control results in blood flow distribution to both the skin and active muscle during exercise (Rowell et al., 1996). Indeed, skin blood flow reaches an apparent upper limit at a *T*_c_ of ∼38°C during exercise, then decreases or plateaus, despite further increase in *T*_c_ (Brengelmann et al., 1977; Kellogg et al., 1993). Based on this, HE might increase microvascular diameter to facilitate thermoregulation, whilst centrally determined increases in flow and vascular density are constrained. These insights require further investigation, which will be facilitated by OCT. The LD flux data we collected would, we propose, misleadingly favour our original hypothesis that HE microvascular responses exceeded those associated with PH.

LDF‐based flux was measured simultaneously at sites adjacent to our OCT probe placement. Although our LDF results indicated that HE induced a greater increase in flux than PH, this difference was not apparent from any of our OCT variables. Blood flux was also modestly correlated with OCT‐generated blood flow in thermoneutral conditions, and an even larger discrepancy was apparent in LH conditions. This disparity might relate to the technical differences between laser Doppler and optical imaging. OCT captures microvessels ∼300 µm beneath the skin (Argarini et al., 2020), whereas laser Doppler signals arise from ∼530 µm depth at rest (Fredriksson et al., 2009) and ∼660 µm during heat stress; (Carter et al., 2016) a limitation of the LDF approach relates to this change in the depth in varying physiological conditions (Carter et al., 2016). LDF might therefore assess capillaries in the papillary plexus at baseline and larger collecting venules post‐heating. In contrast, OCT provides consistent insight into changes that occur in superficial capillaries.

Although we successfully imaged and quantified skin microvessel responses to different thermoregulatory stimuli, this study has some limitations. OCT images were obtained from a small area on the forearm, and we cannot exclude the possibility that these might not be representative of skin regions. Our present OCT algorithms possess relatively limited temporal resolution, and future development will need to resolve this issue if studies are to provide new insights into microvascular mechanisms and effector pathways. In addition, the menstrual phase was documented, but not controlled experimentally in this study, which might contribute to variability in female responses. Fluctuations in ovarian hormones, particularly oestrogen, can influence the skin thermoregulatory response (Brooks et al., 1997; Charkoudian, 2010). Therefore, future studies should control for the menstrual cycle to minimize interindividual variability. Furthermore, this study involved young and healthy volunteers, in whom microvascular and thermoregulation functions are well preserved. Ageing and cardiometabolic disease are known to be associated with both endothelial dysfunction and impaired neurovascular control of skin blood flow (Fuchs et al., 2017; Green et al., 2006; Holowatz et al., 2003; Kenney & Munce, 2003; Minson et al., 2002), which might attenuate the microvascular responses observed in the present study. Extending this approach to older adults and clinical populations would provide valuable mechanistic insight into the microvascular determinants of thermoregulatory insufficiency.

## 5 CONCLUSION

In summary, this is the first study in humans that has imaged, quantified and compared cutaneous microvascular responses to localized skin heating, passive heat exposure and exercise in the heat. Our OCT‐derived results demonstrate that different types of thermal stress induce distinct cutaneous microvascular responses, emphasizing the potential role for OCT in advancing the understanding of microvascular function in humans. Our findings revealed distinct impacts on different OCT parameters, which were not discernible using LDF. Despite being derived from contemporaneous assessments in the same individuals in identical conditions, our OCT and LDF outcome measures provided inconsistent findings that support disparate conclusions.

## AUTHOR CONTRIBUTIONS

Kristanti W. Wigati performed the experiment, conducted data collection, performed data analysis and interpretation, was responsible drafting the manuscript including the preparation of tables and figures for journal submission, and revised and approved the final version of the manuscript. Robert A. McLaughlin contributed to optical coherence tomography methodology and provided technical support, contributed to confirming the OCT‐generated data processing validation, revised and approved the final version of the manusript. Harrison T. Caddy contributed to conseptualisation of the work, performed experiment, conducted data collection, and approved the final version of the manuscript. Howard H. Carter contibuted to conceptualisation of the work, performed the experiment, conducted data collection, and approved the final version of the manusript. Louise H. Naylor contributed to study conception, design, supervision, funding, approval of the final version of the manuscript. Daniel J. Green conceived ther study, invented and developed the technology, supervised students, performed editing, funded the study and approved the final version of the manuscript.

## CONFLICT OF INTEREST

Robert A. McLaughlin is a co‐founder and Director of Miniprobes Pty Ltd, a company that develops optical imaging systems. Miniprobes Pty Ltd did not contribute to or participate in this study. No hardware, software or financial support from the company influenced the acquisition/analysis, and it was confirmed that no proprietary algorithms were used.

## Data Availability

The data support the findings of this study are available from the corresponding author upon reasonable request.
